# Double calcaneal osteotomy for severe adolescent flexible flatfoot reconstruction

**DOI:** 10.1186/s13018-017-0655-3

**Published:** 2017-10-17

**Authors:** Yang Xu, Yong-xing Cao, Xing-chen Li, Yuan Zhu, Xiang-yang Xu

**Affiliations:** 1 Department of Orthopedics, Shanghai Ruijin Hospital North, Shanghai Jiaotong University School of Medicine, Shanghai, China; 2 Department of orthopedics, Shanghai Ruijin Hospital, Shanghai Jiaotong University School of Medicine, Shanghai, 200025 China

**Keywords:** Flatfoot, Osteotomy, Lateral column lengthening, Reconstruction

## Abstract

**Background:**

The timing and strategy of treatment for flatfoot still remain controversial. It is a difficult problem when facing severe adolescent flexible flatfoot because a single procedure cannot realign flatfoot deformity effectively.

**Methods:**

We reviewed 13 adolescent flexible flatfoot patients who underwent double calcaneal osteotomy during May 2012 to June 2015. The mean age of patients was 15.2 ± 1.8 (range, 10–18) years. The American Orthopaedic Foot and Ankle Society Ankle-Hindfoot (AOFAS-AH) scores and SF-36 score were adopted to evaluate the preoperative and postoperative functions of the foot. Changes of hindfoot valgus angles, talonavicular uncoverage angles on AP view and talo-first metatarsal angles, and talar pitch angles and calcaneal pitch angles on the lateral film before and after surgery were measured.

**Results:**

All 13 patients (15 ft) were followed. The mean duration of follow-up was 34.5 ± 15.7 (range, 21–60) months. The hindfoot valgus angle improved from 16.5 ± 4.1 to 2.9 ± 1.6. On the foot AP view, the mean preoperative and postoperative talonavicular coverage angles were 24.9 ± 8.5 and 6.5 ± 3.6. On the lateral view of the foot, the average preoperative and postoperative talo-first metatarsal angles were 18.1 ± 5.5 and 4.9 ± 4.4. The mean preoperative and postoperative talar pitch angles were 36.4 ± 4.7 and 24.0 ± 5.6. The AOFAS-AH score improved from 68.9 ± 12.3 preoperatively to 94.6 ± 3.9 postoperatively.

**Conclusion:**

With additional procedures, double calcaneal osteotomy was an effective method for severe adolescent flexible flatfoot.

## Background

Flatfoot is a common disease, especially in younger populations. Most flatfoot deformities in children are asymptomatic, flexible, and will never require treatment. Flexible flatfoot deformity is characterized by collapse of the medial arch, hindfoot valgus, forefoot abduction, and tightness of the gastrocnemius or gastrocnemius-soleus complex [1]. Contemporary realignment procedures for adolescent flexible flatfoot include subtalar arthroereisis, medial displacement calcaneal osteotomy, spring ligament reconstruction, lateral column lengthening, and arthrodesis. Additional procedures include cotton osteotomy, first tarsometatarsal fusion, and gastrocnemius recession [2].

The forefoot abduction deformity is characterized by an increased talonavicular uncoverage angle, which can be easily treated by a lateral column lengthening procedure. Lateral column lengthening procedures usually contain Evans osteotomy, Mosca osteotomy, and calcaneal Z lengthening osteotomy. But lateral column lengthening is associated with increased lateral plantar pressures and will result in lateral pain or calcaenocuboid arthritis. A combination of lateral column lengthening and medial displacement calcaneal osteotomy will potentially reduce lateral column pressure compared with single lateral column lengthening [3, 4].

The exact indication, time, and methods for adolescent flatfoot reconstruction still remain controversial. Severe flatfoot requires a complex operative plan that contains procedures for both hindfoot and forefoot deformities. In these cases, single procedure such as subtalar arthroereisis usually is not able to correct the deformity. In adolescent patients, avoiding fusion procedure to preserve native hindfoot joint is very important. So, when facing severe flatfoot, double calcaneal osteotomy is a choice to realign the normal foot alignment.

The double calcaneal osteotomy surgery can achieve full correction without the need to fuse any joints. And that is important for young patients. We report the outcomes of double calcaneal osteotomy for severe adolescent flexible flatfoot reconstruction, which was rarely reported before. Limited articles reported the outcomes of double calcaneal osteotomy for treatment of adult flatfoot [5–7], and there is no article reported this method used in adolescent patients. In this study, all patients were older than 14 with hindfoot valgus angle larger than 15° and talonavicular coverage angle larger than 20°.

The goal of this study was to evaluate the outcomes of double calcaneal osteotomy for severe adolescent flexible flatfoot patients. Our hypothesis was that double calcaneal osteotomy was an effective method to deal with such kind of flatfoot.

## Methods

Approval for this retrospective study was obtained from our hospital’s institutional review board. From May 2012 to June 2015, 42 patients operated because of adolescent flexible flatfoot deformity were reviewed. Among these patients, 13 patients with relatively severe flatfoot were treated with double calcaneal osteotomy. The mean age of the patients was (15.2 ± 1.8) years (range, 10 to 18) at the time of operation.

The inclusion criteria were (1) flatfoot without posterior tibial tendon dysfunction, (2) symptomatic flatfoot, (3) flexible flatfoot, (4) older than 14 years old, (5) hindfoot valgus angle larger than 15°, and (6) talonavicular coverage angle larger than 20°.

The exclusion criteria were rigid flatfoot, tarsal coalition, tibial posterior tendon dysfunction flatfoot, congenital vertical talus, and midfoot or hindfoot arthritis.

### Preoperative preparation

Conservative methods including orthotics, custom-fit insoles, and Achilles tendon stretching exercises were recommended before the operation. We usually recommend these treatments for 1 year to observe whether the symptom disappears or the medial arch of the foot appears.

A careful history and clinical examination must be taken before the surgery. The motion of the ankle joint, subtalar joint, and talonavicular joint were recorded. Patients with rigid flatfoot were excluded.

We obtained all patients’ weight-bearing radiographs preoperatively including standard weight-bearing AP and lateral views of the foot, weight-bearing AP views of the ankle, and hindfoot alignment views. CT or MRI scan was performed to identify tarsal coalition or other abnormalities. We used talocalcaneal angle to represent hindfoot valgus angle. The hindfoot alignment view we used in this study was that recommended by Saltzman [8]. The equinus deformity was defined according to the Silfverskiöld test. The isolated gastrocnemius contracture was defined as when the knee was fully extended, the passive ankle dorsiflexion was smaller than 5°. The gastrocnemius-soleus complex contracture was defined as when the knee was in 90° flexion, the ankle dorsiflexion was smaller than 10° [9].

The American Orthopaedic Foot and Ankle Society Ankle-Hindfoot (AOFAS-AH) Score was adopted to determine the functional outcomes of flatfoot patients. The scores are rated as excellent (90 to 100 points), good (80 to 89 points), fair (70 to 79 points), and poor (< 70 points).

### Operative technique

Patients were positioned supine on the operating table under general anesthesia with a thigh tourniquet. First, the Achilles tendon was lengthened by percutaneous Hoke method for the treatment of concomitant equinus deformity [10]. A single oblique incision was made for both medial displacement posterior calcaneal osteotomy and the Evans anterior calcaneal osteotomy. Take care to protect the peroneal tendons. We used the Evans osteotomy to lengthen the lateral column through the anterior part of the calcaneus. The osteotomy was performed vertically 1 cm proximal form the calcaneocuboid joint. A tricortical patella allograft was made wedge-shaped, and the size was determinated according to intraoperative condition. The purpose of the Evans osteotomy was to reduce talonavicular dislocation, which, in the X-rays, was indicated by the talonavicular coverage angle.

After retracting the peroneal tendons upward, dissection was performed to the lateral wall of the calcaneus. A 45° oblique osteotomy was performed with an oscillating saw. The posterior part of calcaneus was removed medially to restore the normal hindfoot alignment. The intraoperative hindfoot alignment was assessed by that when lifting the lower extremity, there was no varus or valgus deformity under direct vision. Most of the Evans osteotomies were fixed with a small plate. All the medial displacement osteotomies were fixed with two titanium alloy compressive hollow screws. If the patient’s calcaneal epiphysis was not closed, we would use two K-wires to fix the calcaneal osteotomy.

**Fig. 1:**
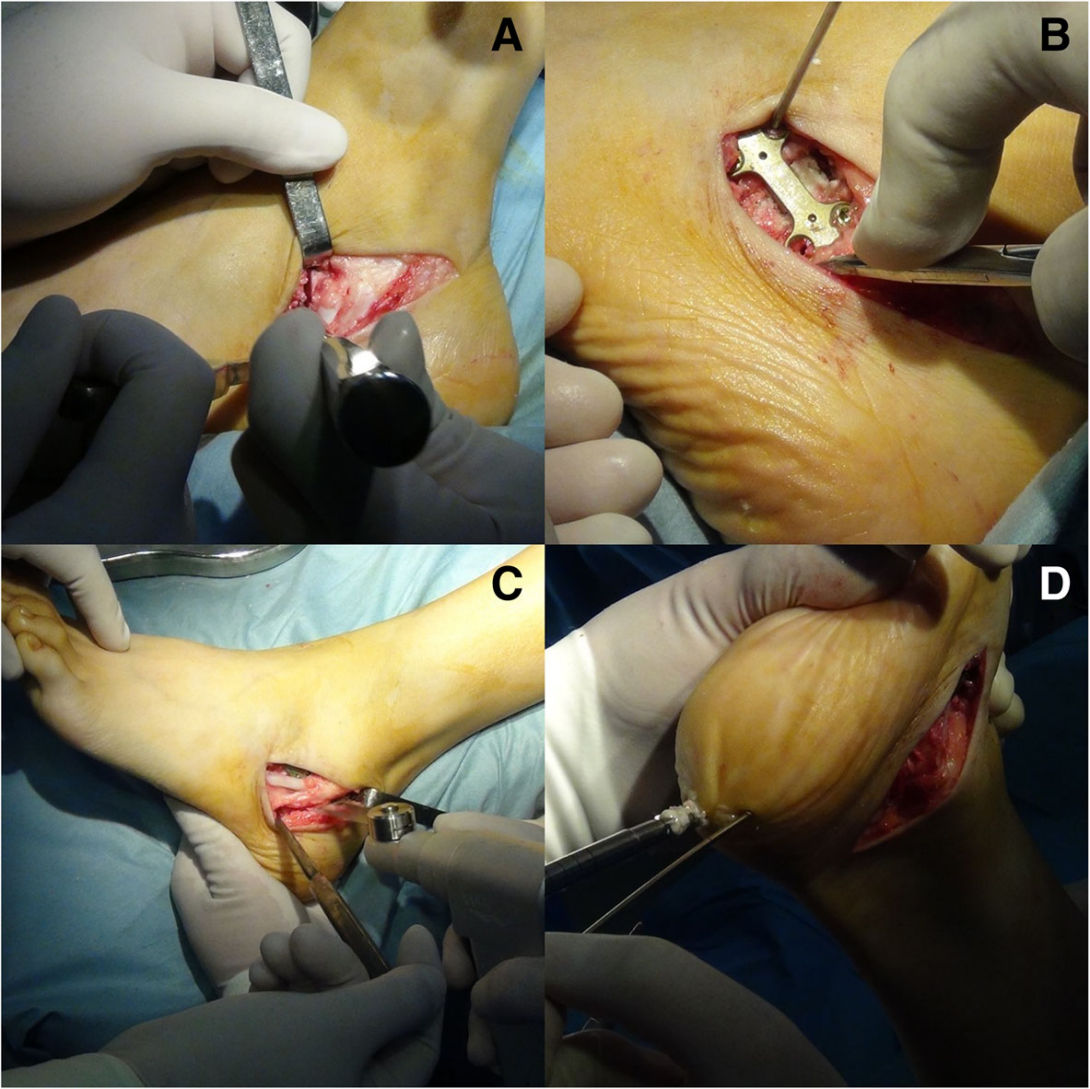
Intraoperative photos. The Evans osteotomy (**a**). The fixation of Evans osteotomy (**b**). The posterior calcaneal osteotomy (**c**). The fixation of calcaneal osteotomy (**d**)

After finishing hindfoot surgery, fixed forefoot supination was managed with a cotton osteotomy through a 2- to 3-cm incision medial to the hallucis longus tendon. The cotton osteotomy was fixed with a small plate. Intraoperative C-arm was used to ensure the adequacy of correction. **(**Fig. 1 **).**

### Postoperative management

After the operation, a boot was recommended to support the osteotomy and patients were kept non-weight-bearing during the initial 6 weeks. In this period, patients were required to do some physical exercises such as exercising the ankle and knee joint. Patients were required to go to outpatient at 6 and 8 weeks postoperatively and take weight-bearing radiographs to estimate the consolidation of osteotomies. The consolidation of the osteotomies and integration of bone graft was at 8 weeks postoperatively. Patients came to outpatient 3 months, 6 months, and 1 year postoperatively to estimate the function of the foot. We recommend internal fixation removal 1 year after surgery.

### Statistic analysis

All analyses were performed with SAS software version 8.1 (SAS Institute Inc., Cary, NC). The results were presented as the mean and standard deviation. The paired Student’s *t* test was used to compare the preoperative and postoperative radiographic measurements and AOFAS-AH scores. Significance was set at *p* < 0.05.

## Results

**Fig. 2:**
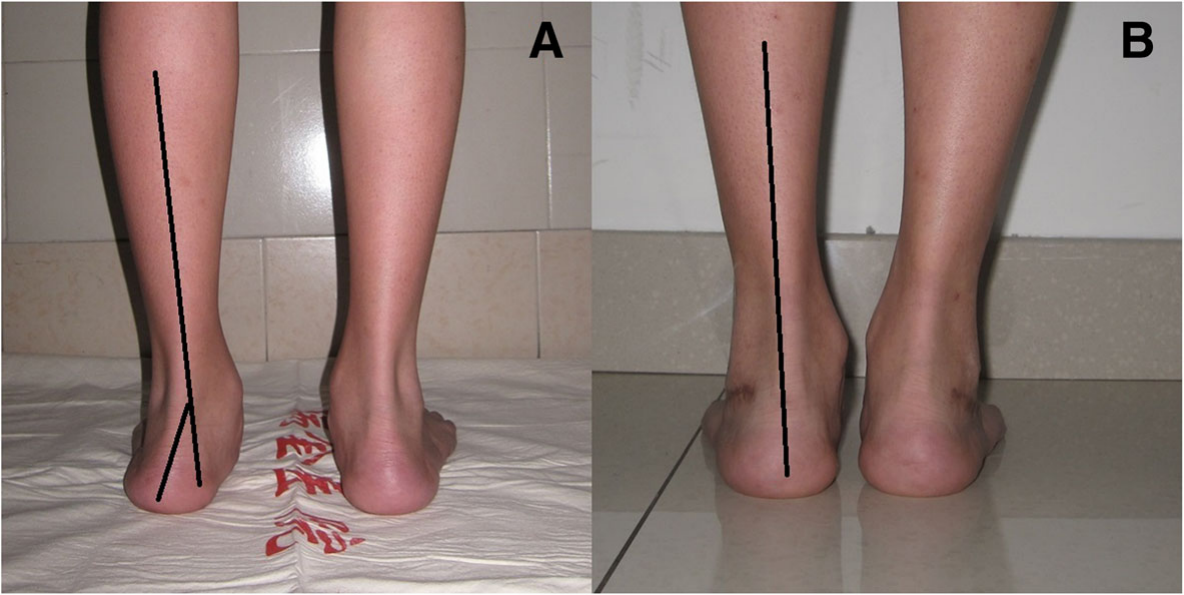
Preoperative (**a**) and postoperative (**b**) photos. The postoperative hindfoot alignment is much better than preoperative alignment

**Fig. 3:**
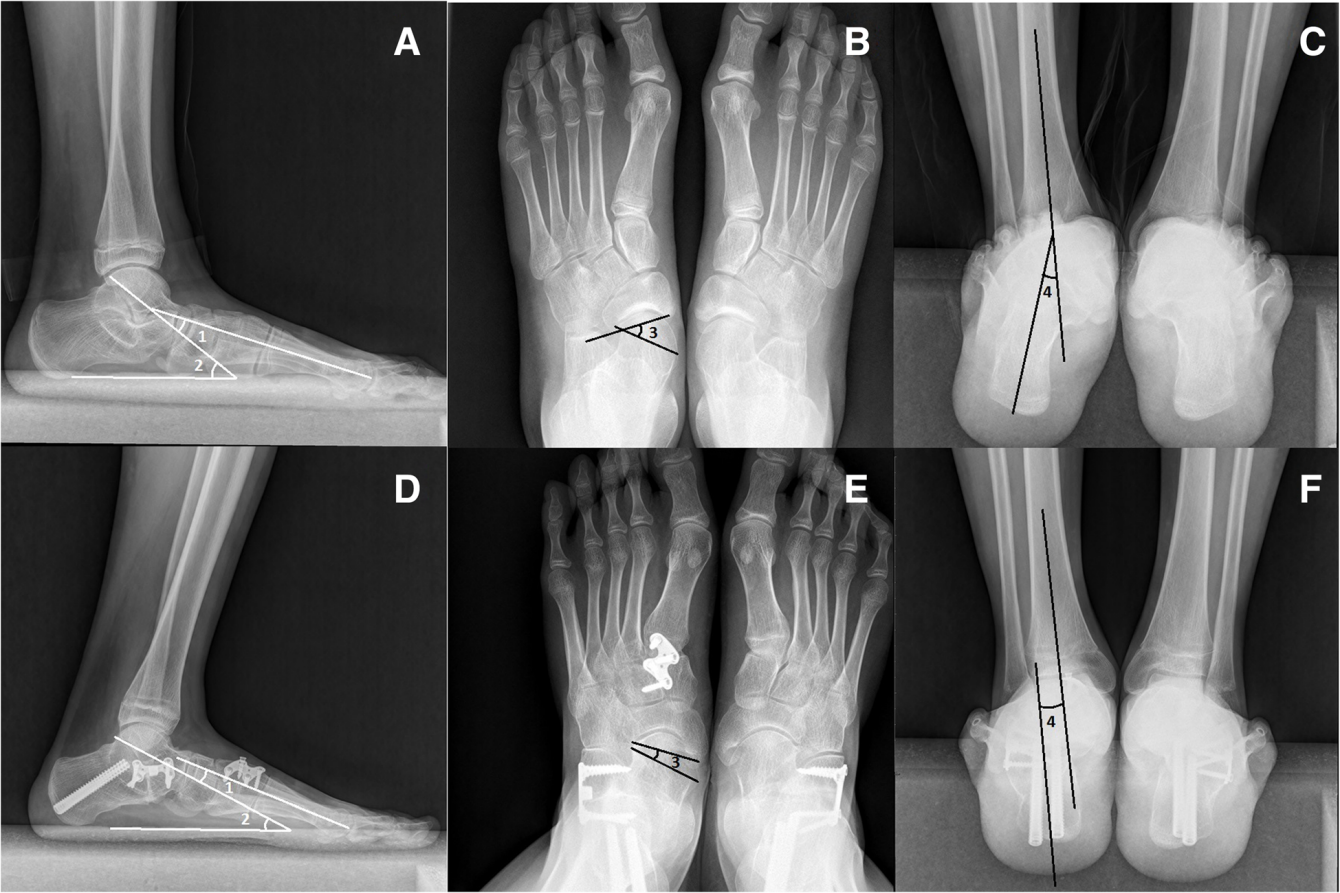
Correction of flexible flatfoot in a 14-year-old boy. Preoperative (**a**–**c**) and postoperative (**d**–**f**) weight-bearing radiographs show the realignment of flatfoot in lateral view, AP view, and hindfoot alignment view. 1, 2, 3, and 4 represent the talo-first metatarsal angle in lateral view, talar pitch angle, talonavicular coverage angle, and hindfoot valgus angle respectively

Thirteen patients were included in this study, including 9 males and 4 females. The average follow-up time was 34.5 ± 15.7 (range, 21 to 60) months. All 15 ft demonstrated clinical and radiographic healing at 8 weeks postoperatively. The hindfoot valgus angle improved from 16.5 ± 4.1 to 2.9 ± 1.6 (*p* < 0.0001). On the foot AP view, the mean preoperative and postoperative talonavicular coverage angle were 24.9 ± 8.5 and 6.5 ± 3.6 (p < 0.0001). On the lateral view of the foot, the average preoperative and postoperative talo-first metatarsal angle were 18.1 ± 5.5 and 4.9 ± 4.4 (*p* < 0.0001). The mean preoperative and postoperative talar pitch angle were 36.4 ± 4.7 and 24.0 ± 5.6 (*p* = 0.0002). The AOFAS-AH score improved from 68.9 ± 12.3 preoperatively to 94.6 ± 3.9 postoperatively (*p* = 0.0002). The SF-36 score improved from 63.7 ± 9.6 to 91.2 ± 5.4 (*p* < 0.0001). (Table 1, Figs. 2 and 3).

**Table 1:** Comparison of preoperative and postoperative variables

	Preoperative	Postoperative	*P* value
Hindfoot valgus angle (°)	16.5 ± 4.1	2.9 ± 1.6	< 0.0001
Talonavicular uncoverage angle (°)	24.9 ± 8.5	6.5 ± 3.6	< 0.0001
Talo-first metatarsal angle in lateral view (°)	− 18.1 ± 5.9	− 4.9 ± 4.4	< 0.0001
Talar pitch angle (°)	36.4 ± 4.7	24.0 ± 5.6	0.0002
AOFAS-AH score	68.9 ± 12.3	94.6 ± 3.9	0.0002

Cotton osteotomy was performed in 9 patients, and 11 patients were performed intramuscular lengthening of the gastrocnemius; the other two were performed Achilles tendon lengthening via percutaneous method.

Two patients felt uncomfortable at the lateral side of the foot, and the symptom disappeared after internal fixation removal. One patient had a superficial infection at the incision, which healed after the usage of oral antibiotic, and this patient had an abnormal gait even after removal of the internal fixation. After systematic rehabilitation, the gait improved a lot. No nonunion or delayed union of the osteotomy was recorded in this study. All patients were satisfied with the surgery.

## Discussion

Flexible flatfoot is a common disease among adolescent patients, which can be symptomatic and lead to impairment of life [11]. Significant controversy exists about the timing and management of adolescent flexible. It is difficult to deal with severe adolescent flexible flatfoot because arthrodesis is not suitable for it and single procedure is not adequate for it. On the other hand, however, flatfoot is a three-dimensional deformity characterized by forefoot abduction, hindfoot valgus, and collapse of the medial foot arch [12]. A combination of biplanar calcaneal osteotomies may work in the treatment of severe adolescent flexible flatfoot.

Lateral column lengthening has been used to deal with flatfoot with prominent forefoot abduction deformity, which is of great significance to the reduction of talonavicular abduction angle [13, 14]. A cadaveric experiment performed by Baxter et al. showed that LCL procedure could correct 60% hindfoot valgus deformity in addition to 100% midfoot abduction deformity [15]. The lateral column lengthening procedure was reported that it was related with increased lateral aspect of foot and calcaneocuboid arthritis [14]. But in our study, no one showed lateral arthritis. Maybe it is because they are adolescents or maybe the sample size is small.

The double calcaneal osteotomy may lead to reduction of lateral column pressure compared with lateral column lengthening alone [3, 4]. And this combination is easy to correct both forefoot abduction and hindfoot valgus deformities simultaneously. The combination of these two osteotomies showed strong ability to restore the hindfoot and midfoot alignment. Furthermore, compared with other procedures, the double calcaneal osteotomy reduces more strain of medial ligament [16]. So, when facing flatfoot patients with large talonavicular angle and hindfoot valgus angle, double calcaneal osteotomy is a good choice to restore the midfoot and hindfoot alignment.

Didomenico et al. reported double calcaneal osteotomy with single, dual-function screw fixation technique [7]. In our study, two osteotomies were fixed separately. The calcaneal medial displacement osteotomy was fixed with two parallel screws, and the Evans osteotomy was fixed with a small plate. We think that it is strong enough to prevent any loss of distraction or displacement of the osteotomies.

In this study, 9 patients underwent cotton osteotomy. Eleven patients underwent intramuscular lengthening of gastrocnemius, and 2 patients underwent percutaneous Achilles tendon lengthening. With concomitant additional surgeries, the alignment of patients’ feet restored much better. In this study, many cases are severe flatfeet that need additional procedures to treat concomitant deformity. But in the treatment of flatfoot, we mainly focus on the alignment of the foot, and in this study, we mainly focus on hindfoot valgus angle and talonavicular coverage angle. Soft tissue surgery would not affect the bone structure obviously. And the cotton osteotomy just affects the talo-first metatarsal angle in the lateral view and has limited effect on hindfoot valgus angle and talonavicular angle.

Adolescences have strong growth ability, so the complications in this study are not so many and all patients achieved good results. But limitations are obvious in this study. First, the sample size is small, which may result in some bias. And the follow-up time is short. Since adolescences are in a constant process of growth and development, it is better to follow up them until they are 18 years old or older. Second, all measurements are based on plain radiographs, which is easily affected by patients’ standing position. We hope that a more specific and accurate measure method will be adopted in the treatment of flatfoot.

## Conclusion

With additional procedures, double calcaneal osteotomy was an effective method for severe adolescent flexible flatfoot. It is a significant method for severe flatfoot alignment reconstruction.

## Abbreviations

AOFAS-AH: The American Orthopaedic Foot and Ankle Society Ankle-Hindfoot scores
SF-36: The Short Form (36) Health Survey
